# Pregnancy-related anxiety symptoms and associated factors amongst pregnant women attending a tertiary hospital in south-west Nigeria

**DOI:** 10.4102/sajpsychiatry.v27i0.1616

**Published:** 2021-03-19

**Authors:** Adesanmi Akinsulore, Akinfenwa M. Temidayo, Ibidunni O. Oloniniyi, Badejoko O. Olalekan, Oladimeji B. Yetunde

**Affiliations:** 1 Department of Mental Health, Faculty of Clinical Sciences, Obafemi Awolowo University, Ife-Ife, Osun State, Nigeria; 2 Department of Obstetrics, Gynaecology and Perimatology, Faculty of Clinical Sciences, Obafemi Awolowo University, Ife-Ife, Osun State, Nigeria

**Keywords:** pregnancy, anxiety, women, gynaecology, Nigeria

## Abstract

**Background:**

Pregnancy can be associated with anxiety symptoms because of anticipated uncertainty.

**Aim:**

This study investigated pregnancy-related anxiety symptoms (PRASs) and their associated factors amongst pregnant women.

**Setting:**

Obafemi Awolowo University Teaching Hospitals Complex, Ile-Ife, Nigeria.

**Methods:**

This cross-sectional survey involved 230 pregnant women attending antenatal clinic at a tertiary teaching hospital in Nigeria. Pregnancy-related anxiety symptoms, maternal worries, personality traits and social support were measured by using Perinatal Anxiety Screening Scale (PASS), Cambridge Worry Scale (CWS), Big Five Personality Inventory (BFI-10) and Maternal Social Support Scale (MSSS) respectively. Socio-demographic and obstetric details were also obtained. The Chi-square, t-test and logistic regression were used.

**Results:**

Respondents’ mean age was 28.2 ± 5.4 years, whilst 192 (83.5%) were of Yoruba ethnicity. Twenty-four respondents (10.4%) were in the first trimester, 85 (37.0%) in the second and 121 (52.6%) in the third trimester. Some 154 (67.0%) were parous. The prevalence of PRAS and major maternal worries were 43.5% and 55.7% respectively. The socio-demographic factors significantly associated with PRAS were age (*p* = 0.004), ethnicity (*p* = 0.001), educational level (*p* = 0.011) and living arrangement (*p* = 0.029). Associated obstetric factors include trimester (*p* = 0.01), hypertension (*p* = 0.006), past miscarriage(s) (*p* = 0.013) and past pregnancy complication (*p* = 0.030). Significant psychosocial factors were partner social support (*p* = 0.038), maternal worries (*p* < 0.001) and extraversion (*p* = 0.016). Factors that contributed significantly to regression models were older maternal age and socio-medical worries.

**Conclusion:**

High level of PRAS and major maternal worries were common amongst antenatal clinic attendees of a tertiary teaching hospital in Nigeria. Older maternal age and socio-medical maternal worries are important predictors of PRAS.

## Introduction

Pregnancy is a significant milestone in the life of a woman and her family. It is a period of mixed feelings in which some pregnant women experience great joy, satisfaction and fulfillment, whilst others may report great stress when dealing with its demands.^1,2^ This great stress may impact the physical, psychological and social health of the mother to be. It is also associated with anxiety symptoms because of the anticipated uncertainty related to pregnancy.^3^ Pregnancy-related anxiety symptoms (PRASs) are worries, concerns and fears about pregnancy, childbirth, infant well-being and future parenting.^4^ Pregnancy-related anxiety symptoms are emotional states that are similar to anxiety but distinct because they are specifically rooted in concerns amongst pregnant women in the context of their pregnancies.^5^

Previous studies on pregnancy anxiety from European and Asian countries have reported a wide range of prevalence rates of 6.8% – 54%.^6,7,8,9^ Nigerian studies on pregnancy anxiety have also reported varying prevalence rates ranging from 7.2% to 39% amongst antenatal women.^10,11,12,13^ However, all these studies explored general pregnancy anxiety by using general anxiety instruments rather than assessing pregnancy-related anxiety. By using an instrument (such as Perinatal Anxiety Screening Scale [PASS]), which specifically measures PRAS, a study conducted amongst pregnant women in India reported a prevalence of 22.6%.^14^ This implies that pregnancy-related anxiety is a common problem amongst pregnant women.

The prevalence of pregnancy-related anxiety varies at different trimesters of pregnancy with high levels reported in the first^1,14^ and third trimesters.^1^ Furthermore, nulliparous women reported higher levels of PRASs than parous women.^1,15^ Other pregnancy-related factors associated with anxiety symptoms are presence of physical illness like hypertension, previous puerperal complications, previous abortion and instrumental delivery, unwanted pregnancy, miscarriages, previous difficult labour or still birth.^1,13,16^ Socio-demographic factors associated with pregnancy anxiety are young maternal age, ethnicity, socio-economic status and level of education.^7,9,17^ Psychosocial factors associated with pregnancy anxiety are maternal worries, personality trait like neuroticism and poor social support.^13,18^

High levels of untreated pregnancy anxiety may have a negative impact on the health and well-being of the pregnant woman and the developing foetus.^19^ Amongst pregnant women, high levels of pregnancy anxiety have been associated with shorter gestation and birth duration miscarriage, and preeclampsia.^20,21,22,23^ Moreover, high levels of pregnancy anxiety have also been associated with preterm birth and low birth weight,^24^ negative emotionality,^25,26^ attention-deficit hyperactivity disorder and developmental delays.^20,27^ Therefore, detecting high levels of PRASs amongst pregnant women attending antenatal clinics is very important for prevention, early intervention and management.

Previously published Nigerian studies on pregnancy anxiety assessed general anxiety amongst pregnant women. However, there is a paucity of data on PRASs amongst Nigerian pregnant women. It is against this background that the present study was designed to determine the prevalence of PRASs amongst Nigerian women attending antenatal clinic in a tertiary hospital and to identify factors associated with those symptoms.

## Methods

### Study design

This study is a facility-based cross-sectional analytical study carried out at the antenatal clinics of a teaching hospital in south-west Nigeria.

### Study setting

The hospital is one of the first-generation teaching hospitals established by the Federal Government of Nigeria, and it provides qualitative health care to people in the south-western part of the country. The antenatal clinics of Department of Obstetrics, Gynecology and Perinatology run 4 days a week with an average of 30 to 40 pregnant women per clinic.

### Study population

The study population comprised pregnant women accessing antenatal services at the Department of Obstetrics, Gynecology and Perinatology of the Obafemi Awolowo University Teaching Hospitals Complex, Ile-Ife, Nigeria. Pregnant women aged 18–45 years and at different trimesters were included in the study. Pregnant women who were critically ill or declined participation were excluded from the study.

### Sample size determination

From the records of the antenatal clinic, the total number of registered pregnant women who accessed professional antenatal care services in the hospital between January and December 2017 was 3458. The sample size of this study was calculated with the formula for total study population of less than 10 000 at 95% confidence level (1.96). Using the estimate of the true proportion of PRASs amongst pregnant women of 17.5% from a previous Nigerian study,^13^ a maximum allowable margin of error of 5%, and allowing 10% for possible incomplete data, a sample size of 230 was obtained.

### Procedure

Pregnant women who met the inclusion criteria were given a copy of the participant information sheet. An average attendance of 30‑40 pregnant women per clinic from the antenatal clinics ensured feasibility for adequate sampling. The aim of the study was explained to each of the pregnant women, and informed consent was obtained from interested participants. Consenting women were recruited into the study, whilst women who refused consent were excluded with no adverse consequence. The participants completed the questionnaire in a convenient room within the antenatal clinic. They were each asked to read through the statements in the questionnaires carefully and then mark what they thought was correct on a rating scale against each statement. Each rating score meaning was explained to them. They first completed the demographic and pregnancy-related variables section of the questionnaire. A questionnaire was used to collect socio-demographic and pregnancy-related variables. Socio-demographic variables obtained were age, marital status, religion, ethnicity, educational level and living arrangement. Maternal age was categorised as 18‑24 years (young mothers), 25–34 years (reference group) and 35 years and above (old mothers). Education was categorised as low (≤secondary school), middle (Nigeria Certificate in Education [NCE] and diplomas) and high (university degree) whilst living arrangement was grouped as living with partner, partner and children, alone and with parents. Pregnancy-related variables obtained were last menstrual period (LMP), duration of pregnancy, wanted pregnancy or not, history of hypertension and diabetes, past history of miscarriage, past history of caesarean section and complications during delivery such as postpartum haemorrhage. The duration of pregnancy was categorised into trimesters.

### Measures

They self-rated their anxiety level by using the PASS, maternal worries by using the Farsi Cambridge Worry Scale (CWS), personality trait by using the 10-item Big Five Personality Inventory (BFI-10) and social support by using the Maternal Social Support Scale (MSSS). Each questionnaire was completed within 15‑20 min.

Pregnancy-related anxiety symptoms were assessed with the PASS.^28^ The PASS is a 31-item self-report questionnaire on a four-point Likert scale (from 0 = ‘not at all’ to 3 = ‘almost always’) with scores ranging from 0 to 93. It measured problematic anxiety in antenatal and postpartum women in four domains that address specific symptoms of anxiety as they appear in perinatal women. These domains form the subscales of the questionnaire which include: (1) Excessive Worry and Specific Fears, (2) Perfectionism, Control and Trauma, (3) Social Anxiety and (4) Acute Anxiety and Adjustment. The PASS was validated for the perinatal period (i.e. during pregnancy or less than 1 year postpartum) in which respondents self-rate each of the four clusters of anxiety symptoms by indicating the frequency of the symptoms over the previous month.^28^ A total PASS score is obtained by adding all of the items on the PASS, and a cut-off score of 26 is recommended to differentiate between high and low risk for PRASs. The Cronbach’s alpha for this scale was 0.96.^28^ Psychosocial factors assessed in this study were maternal worries, personality traits and social support. Maternal worries were measured by using the Farsi CWS.^29^ The original CWS was a 17-item questionnaire that measures worries during pregnancy and contains items such as the infant’s health, financial issues and giving birth.^30^ The Farsi CWS is a 22-item self-report questionnaire on a six-point Likert scale (from 0 = ‘not a worry’ to 5 = ‘major worry’) with scores ranging from 0 and 110. The CWS score was dichotomised into high (4–5, indicating major worry) and low (0–3, signifying less than major worry) scores.^31^ The CWS scale can be used throughout pregnancy and has four-factor structure: (1) socio-medical aspects of having a baby, (2) socio-economic issues, (3) health of baby and (4) health of mother or others and relationships. A higher score reflects higher worries. The Cronbach’s alpha for CWS was 0.886, and subscales were 0.847 for socio-medical, 0.69 for socioeconomic, 0.715 for health of baby and 0.803 for health of mother or others and relationships.^29^

Personality traits were measured by using 10-item BFI-10.^32^ The BFI is a multi-dimensional personality inventory that model’s personality traits and defines five relatively distinct areas of individual differences: openness to new experiences; extraversion; agreeableness; conscientiousness and neuroticism. The BFI-10 was rated on a five-point Likert scale (from 1= ‘disagree strongly’ to 5 = ‘agree strongly’). Two BFI items were selected for each Big Five dimension. Cronbach’s alpha for the distinct area of individual differences were openness (0.79), conscientiousness (0.82), extraversion (0.89), agreeableness (0.74) and neuroticism (0.86).^32^ The scoring procedure of this scale indicated that the higher the score above the global means score on each subscale, the higher the individual’s traits on that particular personality factor and vice-versa.

Social support was measured by using the MSSS.^33^ It is a 6-item questionnaire rated on a 5-point Likert scale (from 1 = ‘never’ to 5 = ‘always’). The six questions inquire about how much support the woman feels she receives from friends, family and partner. One question inquired about social support from friends and family, whilst four questions for partner of which two of the questions were reverse scored. For this study, the mean scores for friend, family and partner were reported. The Cronbach’s alpha for MSSS was 0.82.^33^

### Data analysis

The IBM-SPSS version 21.0 was used for statistical analysis. Descriptive statistics such as frequency and percentages were used to describe categorical data, whilst mean with standard deviation (SD) was used to describe continuous data. Inferential statistics made use of Chi-square for the associations of PRASs with demographic and pregnancy-related variables. Independent t-test was used to compare the mean scores of the psychosocial factors (CWS, BFI-10 and MSSS) amongst pregnant women with low and high PRASs. Hierarchical logistic regression analysis with the enter method was used to explore the predictors (socio-demographic and pregnancy-related characteristics as well as psychosocial factors) of PRASs. The dependent variable was PRAS categorised as low and high. The independent variables included were the socio-demographic and pregnancy-related factors, as well as the psychosocial factors. Three blocks and significant variables at the bivariate level were entered into the model. Socio-demographic variables (age group, educational level, living arrangement and ethnicity) were first entered into Model 1, then pregnancy-related factors (trimester, hypertension, past miscarriage and past pregnancy complication[s]) were added to Model 2 and finally psychosocial factors (maternal worries and its subscales, social support from partners and extraversion) were added to Model 3. All statistical tests were two-tailed, and the level of significance was *p*-value less than 0.05.

### Ethical considerations

This study was approved by the Ethics and Research Committee of a teaching hospital in south-west Nigeria (Protocol number: ERC/2018/04/11). Written informed consent was obtained from all respondents after the aim and objectives of the study were explained to them by using respondent’s information sheet. The issues of confidentiality and voluntariness in participation were also explained.

## Results

The prevalence of PRASs was 43.5%, and 40 respondents (17.4%) reported severe symptoms. The PRAS subscale with the highest mean score was perfectionism, control and trauma (1.15 ± 0.66), whilst social anxiety subscale had the lowest (0.62 ± 0.66) (Table 1). One hundred and forty-five respondents (63.1%) were aged 25‑34 years old, whilst 30 respondents (13.0%) were 35 years and above with a mean age of 28.2 ± 5.4 years. One hundred and eighty respondents (78.3%) were married, 165 (71.7%) were Christians and 198 (83.5%) were of Yoruba ethnicity. Eighty-five had middle level education (37.0%) and 80 (34.7) had low level education. One hundred and twenty-four respondents (53.9%) were living with partner and children, whilst only seven (3.0%) lived alone. Socio-demographic factors significantly associated with high level PRAS were maternal age (*p* = 0.004), ethnicity (*p* = 0.001), educational level (*p* = 0.011) and living arrangement (*p* = 0.029) (Table 2). One hundred and twenty-one respondents (52.6%) were in the third trimester, whilst 24 respondents (10.4%) were in the first trimester. One hundred and fifty-four respondents (67.0%) were parous, and 210 (91.3%) expressed that the pregnancy was wanted. Fourteen respondents (6.1%) had hypertension, whilst eight respondents had diabetes. Only 41 respondents (17.8%) experienced a miscarriage in a previous pregnancy, whilst 27 (11.7%) had experienced pregnancy complications. One hundred and twenty-eight respondents reported major maternal worries. Pregnancy-related factors significantly associated with high level of PRAS were trimester (*p* = 0.01), comorbid hypertension (*p* = 0.006), past miscarriages (*p* = 0.013), past pregnancy complication (*p* = 0.03) and maternal worries (*p* < 0.001) (Table 3). The maternal social support with highest mean score was family social support (4.61 ± 0.73), and partner social support had the lowest mean score (3.71 ± 0.69). Maternal worries subscale with the highest mean score was socio-medical (1.26 ± 1.00) whilst socio-economic subscale had the lowest mean score (0.97 ± 0.91). Personality trait with the highest mean score was conscientiousness (3.56 ± 1.02) and neuroticism had the lowest mean score (2.77 ± 0.95). Significant psychosocial factors were partner social support (*p* = 0.038), all the subscales of maternal worries (*p* < 0.001) and extroversion (*p* = 0.016) (Table 4).

**TABLE 1:** Prevalence, severity and subscales of pregnancy-related anxiety symptoms.

Variables	Frequency		Mean	±SD
	*n*	%		
**Prevalence**				
Low PRASs (0–25)	130	56.5	-	-
High PRASs (26–93)	100	43.5	-	-
**Severity of symptoms**				
Asymptomatic (0–20)	96	41.7	-	-
Mild to moderate (21–41)	94	40.9	-	-
Severe (42–93)	40	17.4	-	-
**Subscales**				
Excessive worry	-	-	0.78	0.66
Perfectionism, control and trauma	-	-	1.15	0.63
Social anxiety	-	-	0.62	0.66
Acute anxiety and adjustment	-	-	0.70	0.63

PRASs, pregnancy-related anxiety symptoms; SD, standard deviation.

**TABLE 2:** Socio-demographic factors in association with pregnancy-related anxiety symptoms amongst respondents.

Variables	Total *n* = 230		Pregnancy-related anxiety symptoms				Statistics
			Low *n* = 130 (56.5%)		High *n* = 100 (43.5%)		
	*n*	%	*n*	%	*n*	%	
**Age group**							
18–24	55	23.9	24	43.6	31	56.4	*χ*^2^ = 11.12
25–34	145	63.1	94	64.8	51	35.2	*p* = 0.004
≥ 35	30	13.0	12	40.0	18	60.0	
**Marital status**							
Married	180	78.3	23	46.0	27	54	*χ*^2^ = 2.88
Not married	50	21.7	107	59.4	73	40.6	*p* = 0.09
**Religion**							
Christianity	165	71.7	97	58.8	68	41.2	*χ*^2^ = 1.22
Islam	65	28.3	33	50.8	32	49.2	*p* = 0.269
**Ethnicity**							
Yoruba	192	83.5	118	61.5	74	38.5	*χ*^2^ = 11.52
Others	38	16.5	12	31.6	26	68.4	*p* = 0.001
**Educational level**							
Low	80	34.7	35	43.8	45	56.3	*χ*^2^ = 9.03
Middle	85	37.0	51	60.0	34	40.0	*p* = 0.011
High	65	28.3	44	67.7	21	32.3	
**Living arrangement**							
Partner	86	37.4	56	65.1	30	34.9	*χ*^2^ = 8.99*
Partner and children	124	53.9	67	54.0	57	46.0	*p* = 0.029
Parents	13	5.7	3	23.1	10	76.9	
Alone	7	3.0	4	57.1	3	42.9	

* , Likelihood ratio applied.

**TABLE 3:** Pregnancy-related factors in association with pregnancy-related anxiety symptoms amongst respondents.

Variables	Total *n* = 230 (%)		Pregnancy-related anxiety symptoms				Statistics
			Low *n* = 130 (56.5%)		High *n* = 100 (43.5%)		
	*n*	%	*n*	%	*n*	%	
**Trimester**							
First	24	10.4	15	62.5	9	37.5	*χ*^2^ = 9.29
Second	85	37.0	37	43.5	48	56.5	*p* = 0.01
Third	121	52.6	78	64.5	43	35.5
**No. of pregnancy**							
Primigravida	76	33.0	44	57.9	32	42.1	*χ*^2^ = 0.087
Multigravida	154	67.0	86	55.8	68	44.2	*p* = 0.768
**Pregnancy wanted**							
Yes	210	91.3	120	57.1	90	42.9	*χ*^2^ = 0.379
No	20	8.7	10	50.0	10	50.0	*p* = 0.538
**Hypertension**							
Yes	14	6.1	3	21.4	11	78.6	*χ*^2^ = 7.47
No	210	93.9	127	58.8	89	41.2	*p* = 0.006
**Diabetes**							
Yes	8	3.5	4	50.0	4	50.0	*χ*^2^ = 0.143
No	222	96.5	126	56.8	96	43.2	*p* = 0.705
**Miscarriages**							
Yes	41	17.8	16	39.0	25	61.0	*χ*^2^ = 6.216
No	189	82.2	114	60.3	75	39.7	*p* = 0.013
**Pregnancy complications**							
Yes	27	11.7	10	37.0	17	63.0	*χ*^2^ = 4.726
No	203	88.3	120	59.1	83	40.9	*p* = 0.03
**Maternal worries**							
Less major worry	102	44.3	79	77.5	23	22.5	*χ*^2^ = 32.67
Major worry	128	55.7	51	39.8	77	60.2	*p* ≤ 0.001

**TABLE 4:** Comparison of mean scores of psychosocial variables between respondents with low and high pregnancy-related anxiety symptoms.

Variables (range)	Total		PRASs				Statistics
			Low		High		
	Mean	±SD	Mean	±SD	Mean	±SD	
**MSSS**							
Friend (1– 5)	4.20	1.11	4.12	1.18	4.30	1.00	*t* = -1.198, *p* = 0.232
Family (1–5)	4.61	0.73	4.66	0.70	4.55	0.77	*t* = 1.147, *p* = 0.253
Partner (1–5)	3.71	0.69	3.79	0.72	3.60	0.64	*t* = 2.089, *p* = 0.038
**CWS**							
Socio-medical (0–4.11)	1.26	1.00	0.83	0.74	1.81	1.03	*t* = -8.391, *p* ≤ 0.001
Socioeconomic (0–4.33)	0.97	0.91	0.65	0.73	1.39	0.96	*t* = -6.694, *p* ≤ 0.001
Health of baby (0–5.0)	1.14	1.23	0.79	1.11	1.60	1.23	*t* = -5.241, *p* ≤ 0.001
Health of MOR (0–4.0)	1.04	1.06	0.68	0.80	1.50	1.18	*t* = -6.290, *p* ≤ 0.001
**Personality traits**							
Extraversion (1–5)	3.17	1.06	3.02	1.04	3.36	1.07	*t* = -2.433, *p* = 0.016
Agreeableness (1–5)	3.40	1.08	3.49	1.06	3.28	1.09	*t* = 1.486, *p* = 0.139
Conscientiousness (1–5)	3.56	1.02	3.51	1.05	3.61	0.99	*t* = -0.759, *p* = 0.448
Neuroticism (1–5)	2.77	0.95	2.67	0.92	2.87	0.98	*t* = -1.685, *p* = 0.093
Openness (1–5)	3.18	0.91	3.20	0.99	3.14	0.80	*t* = 0.452, *p* = 0.651

MOR, mother/others & relationships; MSSS, Maternal Social Support Scale; CWS, Cambridge Worry Scale; PRASs, pregnancy-related anxiety symptoms; SD, standard deviation.

Table 5 shows the hierarchical logistic regression model. Model 1 containing socio-demographic predictors was statistically significant, *χ* (8, *N* = 230) = 32.30, *p* < 0.001, indicating that the model was able to distinguish between respondents with high and low PRASs. The model as a whole explained between 13.1% (Cox and Snell *R*-square) and 17.6% (Nagelkerke *R*-square) of the variance in PRAS level, and correctly classified 66.5% of cases. Only three of the independent variables made a unique statistically significant contribution to Model 1 (older women, living with parents and other tribes). In Model 2, pregnancy-related variables were added, and it was statistically significant, *χ* (13, *N* = 230) = 45.87, *p* < 0.001, indicating that the model was able to distinguish between respondents with high and low PRASs. The model as a whole explained between 18.1% (Cox and Snell *R*-square) and 24.2% (Nagelkerke *R*-square) of the variance in PRAS level, and correctly classified 69.6% of cases. Only two of the independent variables made a unique statistically significant contribution to Model 2 (second trimester pregnancy and other tribes). In Model 3, psychosocial variables were added, and it was statistically significant, *χ* (20, *N* = 230) = 96.51, *p* < 0.001, indicating that the model was able to distinguish between respondents with high and low PRASs. The model as a whole explained between 34.3% (Cox and Snell *R*-square) and 46.0% (Nagelkerke *R*-square) of the variance in PRAS level, and correctly classified 81.3% of cases. Only two of the independent variables made a unique, statistically significant contribution to Model 3 (being 35 years and above and socio-medical maternal worries).

**TABLE 5:** Hierarchical logistic regression analysis of predictors of pregnancy-related anxiety symptoms amongst respondents.

Variables	Model 1		Model 2		Model 3	
	OR	95% CI	OR	95% CI	OR	95% CI
**Age group (years)**						
18–24	1.863	0.888–3.906	1.678	0.759–3.709	1.453	0.586–3.604
25–34 (ref)	1.000	-	1.000	-	1.000	-
≥ 35	2.521*	1.081–5.883	2.148	0.867–5.323	3.080*	1.102–8.608
**Education level**						
Low	1.781	0.852–3.723	1.709	0.795–3.675	1.761	0.701–4.423
Middle	1.198	0.583–2.463	1.061	0.501–2.247	0.710	0.295–1.708
High (ref)	1.000	-	1.000	-	1.000	-
**Living with**						
Partner (ref)	1.000	-	1.000	-	1.000	-
Partner and children	1.521	0.814–2.841	1.389	0.722–2.671	1.257	0.279–7.919
Parents	4.396*	1.013–19.074	4.413	0.987–19.73	1.486	0.578–2.733
Alone	0.955	0.179–5.097	1.081	0.177–6.614	0.504	0.052–4.860
**Ethnicity**						
Yoruba (ref)	1.000	-	1.000	-	1.000	-
Others	3.644**	1.661–7.994	3.050**	1.357–6.852	2.467	0.940–6.474
**Trimester**						
First	-	-	0.974	0.351–2.700	0.545	0.158–1.887
Second	-	-	1.936*	1.033–3.627	1.297	0.621–2.710
Third (ref)	-	-	1.000	-	1.000	-
**Hypertension**						
No (ref)	-	-	1.000	-	1.000	-
Yes	-	-	3.143	0.694–14. 239	5.034	0.883–28.700
**Miscarriage**						
No (ref)	-	-	1.000	-	1.000	-
Yes	-	-	2.135	0.979–4.657	1.355	0.555–3.304
**Pregnancy complication**						
No (ref)	-	-	1.000	-	1.000	-
Yes	-	-	1.509	0.552–4.123	1.090	0.356–3.338
**Maternal worries**						
Less than major worries (ref)	-	-	-	-	1.000	-
Major worries	-	-	-	-	1.618	0.667–3.928
**Maternal worries Subscales**						
Socio-medical	-	-	-	-	2.802**	1.409–5.573
Socioeconomic	-	-	-	-	1.134	0.576–2.230
Health of baby	-	-	-	-	1.001	0.667–1.502
Health of mother or others and relatives	-	-	-	-	0.941	0.530–1.673
Social support partner	-	-	-	-	0.574	0.329–1.002
Extraversion	-	-	-	-	1.216	0.856–1.725

CI, class interval; OR, odds ratio.

* , *p* < 0.05;

** , *p* < 0.01.

## Discussion

This study provides useful information about the prevalence of PRASs and their associated factors amongst a sample of Nigerian pregnant women. The results of this study show that pregnancy-related anxiety is a common disorder amongst pregnant women. Also, the consequence of experiencing pregnancy-related anxiety on the health and well-being of both the pregnant woman and the developing foetus calls for investigation into the factors that are associated with it during the course of pregnancy.

The prevalence rate of 43.5% of PRASs reported in this study is within the range reported in previous studies carried out in different socio-economic and cultural settings.^8,31^ It is noteworthy that the prevalence of pregnancy-related anxiety in this study was close to the prevalence of anxiety disorder in pregnancies reported in a Nigerian study.^10^ It is lower than that observed in an Indian study amongst pregnant women at less than 24 weeks,^34^ whilst it was higher than what was reported in Tanzania.^35^ The differences in the prevalence rates could be attributed to different measuring instruments used, as well as the socio-demographic and cultural diversity of the study population.

Amongst the socio-demographic factors, a significant association of PRASs with maternal age, respondent’s level of education, living arrangement and ethnicity was demonstrated. In this study, high levels of PRASs were found amongst the younger and older women with a U pattern. Some studies show that younger maternal age is associated with higher levels of pregnancy-related anxiety,^1,18^ whilst another study reported older maternal age.^36^ Also, one study did not report any association between pregnancy-related anxiety and maternal age.^37^ The younger women, especially those who were pregnant for the first time, had not experienced pregnancy and childbirth before and did not know what to expect. This may be responsible for their high pregnancy-related anxiety. On the other hand, amongst those older women with poor obstetric history, the fear of outcome of pregnancy may be responsible for their high pregnancy anxiety levels. With regard to the association between pregnancy-related anxiety and women’s level of education, women with low levels of education had highest proportion with high levels of pregnancy-related anxiety. Pregnant women living with their parents had a higher proportion with high levels of pregnancy-related anxiety, whilst those living with their partner reported that they had the lowest proportion with high pregnancy-related anxiety. This is quite understandable, as the support provided by the partner may help them to cope with the stresses of pregnancy.

The burden of pregnancy-related anxiety was apparently higher amongst women who belonged to the other tribes. This is similar to the findings of a previous study that found significant association between pregnancy anxiety and not speaking the predominant language.^38^ Although English is the official language of communication in Nigeria, the predominant language spoken in our study setting is Yoruba. Therefore, pregnant women especially those with low levels of education who do not speak Yoruba language may have more difficulty communicating with healthcare workers, or they may experience stigma as a result of their inability to speak the predominant language, leading to higher pregnancy anxiety.

Paradoxically, in the present study, a higher proportion of pregnant women with high levels of PRASs were observed in the second trimester. This is in contrast with what was reported in previous studies that observed low levels of pregnancy-related anxiety in the second trimester.^8,21^ In this study, we did not assess when the pregnant women registered for antenatal care; previous Nigerian studies have reported that most of them registered during the second trimester.^39,40^ They are given health education about the expected changes in pregnancy and risk factors to adverse pregnancy outcomes and these may make them worry, thereby possibly increasing pregnancy-related anxiety during this period.

Although previous studies have reported higher levels of pregnancy-related anxiety amongst primigravida, this study could not demonstrate any significant association. Also, unwanted pregnancy and history of diabetes were not significantly associated with pregnancy-related anxiety. In this study, pregnant women with comorbid hypertension, past history of miscarriage and complicated pregnancy reported higher burden of pregnancy-related anxiety.

Amongst the psychosocial factors, maternal worries, extroversion and social support from partners were significant factors associated with pregnancy-related anxiety in this study. In this study, 55.7% had ‘a major worry’ (scoring 4–5) about their pregnancy, and this is significantly higher than what was reported in a previous study.^31^ Personality traits are constant patterns of thought, emotion and behaviour that describe an individual across time and events.^13^ Neuroticism reflects emotional stability, anxiety and impulse control. Women whose personality is characterised by a high level of neuroticism are more likely to experience pregnancy anxiety.^13,41^ In our study, although women with pregnancy-related anxiety reported higher levels of neuroticism, it was not statistically significant. Extraversion examines sociability, fluency and assertiveness. In this study, extraversion was associated with pregnancy-related anxiety. Possible explanation is that extraversion is associated with assertions, which may enable women to express themselves better.

Increased perceived partner social support appears to reduce the risk for pregnancy-related anxiety, as reported in this study. This is similar to previous research that found significant associations between pregnancy-related anxiety and low social support.^42,43^ Although this study reported a significant association between pregnancy anxiety and partner social support that was in the expected direction, these findings were not significant in the multivariate analyses. This suggests that other factors, often confounded with partner social support, are more powerful at the multivariate level. This is consistent with the results of a previous study.^38^ The predictors of pregnancy-related anxiety in the multivariate analysis in this study were older maternal age and socio-medical maternal worries.

The results of this study need to be interpreted in the light of some limitations. Firstly, the use of self-administered questionnaires may introduce some biases such as social acceptability and recall bias. Also, the study design is cross-sectional in nature, so temporality of studied variables could not be established. Therefore, longitudinal studies are needed to know how pregnancy-related anxiety changes across the course of pregnancy. Moreover, the study was conducted in a single centre, predominantly in a Yoruba setting; the results of this study may have limited generalisability to pregnant women from other Nigerian culture. Despite these limitations, the strength of this study lies in the use of standardised instruments to measure pregnancy-related anxiety and other factors amongst the pregnant women.

## Conclusion

High levels of PRASs and major maternal worries were common amongst a sample of pregnant women attending a tertiary hospital in south-west Nigeria. The predictors of PRASs were older maternal age and socio-medical worries. The results of this study have significant implications for both primary and secondary prevention efforts. There is the need for integrated routine screening of PRASs during routine antenatal care. Early detection, prevention and appropriate management of PRASs may help women to cope with the challenges of pregnancy.
